# Correction: Yang et al. Engineering n-Type and p-Type BiOI Nanosheets: Influence of Mannitol on Semiconductor Behavior and Photocatalytic Activity. *Nanomaterials* 2024, *14*, 2048

**DOI:** 10.3390/nano16020115

**Published:** 2026-01-15

**Authors:** Shuo Yang, Wenhui Li, Kaiyue Li, Ping Huang, Yuquan Zhuo, Keyan Liu, Ziwen Yang, Donglai Han

**Affiliations:** 1 School of Materials Science and Engineering, Changchun University, Changchun 130022, China; 240501286@mails.ccu.edu.cn (W.L.); 220502210@mails.ccu.edu.cn (K.L.); 230502246@mails.ccu.edu.cn (P.H.); 240501261@mails.ccu.edu.cn (Y.Z.); 2 Laboratory of Materials Design and Quantum Simulation College of Science, Changchun University, Changchun 130022, China; 3 School of Materials Science and Engineering, Changchun University of Science and Technology, Changchun 130022, China; 2022101221@mails.cust.edu.cn (K.L.); 2024101323@mails.cust.edu.cn (Z.Y.)

In the original publication [1], reference [22] was retracted prior to this publication. Concerns were raised on this matter. The authors would like to delete the reference:

“Ahmad, A.; e Noor, A.; Anwar, A.; Majeed, S.; Khan, S.; Nisa, Z.U.; Ali, S.; Gnanasekaran, L.; Rajendran, S.; Li, H. Support based metal incorporated layered nanomaterials for photocatalytic degradation of organic pollutants. *Environ. Res*. **2024**, *260*, 119481.”

With this correction, the order of some references has been adjusted accordingly. The authors state that the scientific conclusions are unaffected. The reference order has been adjusted accordingly. This correction was approved by the Academic Editor. The original publication has also been updated.
